# 3D-Printed Customized Arch-Support Insoles Improve Gait Mechanics and Ankle Alignment in Young Adults with Functional Flat Foot During Uphill Walking

**DOI:** 10.3390/medicina61020281

**Published:** 2025-02-06

**Authors:** Sanghee Park, Jin-Hwa Jung, Shi Lei, Eui-Young Jung, Hwi-Young Cho

**Affiliations:** 1 Department of Exercise Rehabilitation, Gachon University, Incheon 21936, Republic of Korea; sangheepark1@gachon.ac.kr; 2 Department of Occupational Therapy, Semyung University, Jecheon 27136, Republic of Korea; otsalt@semyung.ac.kr; 3 Department of Physical Therapy, Gachon University, Incheon 21936, Republic of Korea; shilei1997@gachon.ac.kr (S.L.); noel950@gachon.ac.kr (E.-Y.J.)

**Keywords:** functional pes planus, flat foot, 3D-printed insole, propulsive phase, uphill walking

## Abstract

*Background and Objectives*: Weight-bearing activities exacerbate pain and fatigue in functional flat foot, with uphill walking presenting additional challenges due to increased external loads. The current study investigates whether 3D-printed customized arch-support insoles can enhance gait variables and ankle alignment during uphill walking. *Materials and Methods*: Twenty healthy young adults, divided into two groups (normal foot condition (control, *n* = 10), functional flat foot (FF, *n* = 10)), walked on a treadmill at a 10% incline under two conditions: wearing shoes alone (shoe) or wearing shoes with 3D-printed customized arch-support insoles (SI). Gait pattern, center of force (COF), and ankle joint angles were analyzed by OptoGait, Tekscan, and Kinovea, respectively. *Results*: The foot flat phase of the gait pattern was prolonged in individuals with FF compared to the control under both shoe and SI conditions, whereas the propulsive phase was shortened with the SI. Medial deviation of the COF during the propulsive phase, observed in individuals with FF under the shoe condition, was corrected to a more lateral alignment with the SI, resembling the COF alignment of the control. Additionally, individuals with FF under the shoe condition exhibited increased ankle pronation compared to the control, whereas the SI moderated pronation, achieving alignment closer to that of the control. *Conclusions*: These findings indicate that the 3D-printed customized arch-support insoles can improve gait mechanics and ankle alignment in individuals with FF, particularly under challenging conditions such as uphill walking.

## 1. Introduction

Functional pes planus, commonly referred to as flat foot (FF), is characterized by a collapsed or lowered medial longitudinal arch during weight-bearing activities, while the arch height is restored under non-weight-bearing conditions (e.g., during rest) [1]. This condition disrupts normal foot biomechanics, often resulting in excessive inward rolling of the foot (overpronation), which causes hindfoot valgus, midfoot abduction, and forefoot supination [2]. The misalignments are closely associated with an increased susceptibility to injuries, including plantar fasciitis [3], ankle sprain [4], and chronic foot pain or fatigue [5]. Consequently, the misalignments place greater stress and instability on the ankle joint and lower leg muscles, potentially causing discomfort, reduced mobility, and an elevated risk of injury throughout the lower-limb kinetic chain.

To manage the symptoms of FF, various intervention strategies have been developed to support the foot arch and improve ankle alignment such as insoles and arch supports. Arch supports designed with 3D printing technology, in particular, offer a notable advantage by providing a customized fit, ensuring superior support compared to traditional mass-manufactured orthoses [6,7,8]. Previous studies have demonstrated that 3D-printed insoles and foot orthoses alleviate abnormal pressure, redistribute loads, and reduce foot pain in individuals with musculoskeletal conditions such as asymptomatic or symptomatic FF and joint osteoarthritis [6,9,10]. Foot orthoses have also been associated with improved ankle alignment by reducing peak ankle eversion angles and moments [7,10]. Additionally, a greater contact area in orthoses has been linked to enhanced lower-limb balance, contributing to improved gait stability [11,12]. These findings suggest that the stability provided by customized orthoses can play a significant role in improving gait mechanics in individuals with FF.

However, evaluations of the effectiveness of FF orthoses have primarily focused on level walking, measuring changes in muscle activation [13], gait mechanics [14], and joint angles [7] (e.g., kinetics and kinematics in the gait cycle). Jiang et al. demonstrated that individuals with FF exhibit increased pressure in the middle foot area during uphill walking due to the rise in vertical ground reaction force [15], which negatively impacts ankle and knee alignment, contributing to foot shape alternations and accelerating the progression to rigid flat foot [16]. Furthermore, an imbalance in plantar pressure distribution observed in individuals with FF has been associated with increased pain and fatigue [17]. These findings suggest that uphill walking exacerbates gait mechanic challenges for individuals with FF. In real-life scenarios, most daily activities take place on uneven surfaces, making uphill walking particularly problematic for individuals with FF compared to those with normal arches. These challenges arise from an increased load and stretching of the plantar fascia, as well as mechanical overload on the metatarsals during the stance phase. Such mechanical stresses lead to transient compensatory changes in gait patterns, joint alignment, and muscle activation, further increasing plantar fascia stress in individuals with FF. Despite these insights, limited research has investigated how customized orthoses influence gait kinetics and kinematics in different environmental contexts, such as incline walking.

It remains unclear whether 3D-printed customized orthoses designed to enhance stability can improve the gait variables impaired by FF during uphill walking. Therefore, in the current study, we hypothesize that 3D-printed customized arch-support insoles, specifically designed to increase stability in the midfoot and rearfoot areas, will significantly improve the gait mechanics and ankle alignment in young adults with functional pes planus during uphill walking.

## 2. Materials and Methods

### 2.1. Subject Information

A total of 20 young adults (10 males, 10 females) with or without FF were recruited. An informed consent form was provided to all subjects before participating in the current study at the initial visit. The inclusion criteria included: (1) arch appeared during standing on tiptoe and (2) Foot Posture Index (FPI) total score of ≥6 for the FF group, which was assessed in the standing position. The FPI was based on 6 items covering (1) talar head palpation, (2) the curvature above vs. below the lateral malleoli, (3) calcaneal inversion vs. eversion, (4) the level of the medial arch height, (5) talonavicular congruence, and (6) forefoot abduction vs. adduction with respect to the rearfoot (1, 2, 3), the midfoot (4) and the forefoot (5, 6). The scores of each factor ranged from −2 to +2 (−2 for clear signs of supination, 0 for neutral, and +2 for clear signs of pronation). The final scores were summed, which ranged from −12 to +12. FF was considered to fall within the +6 to +10 range. Exclusion criteria included experience with foot orthosis usage within a month, a surgical history of a foot (including the ankle) fracture/reconstruction/osteotomy or a relevant soft-tissue injury, or an inability to complete the gait tests. Ethical approval for the current study was obtained from the Gachon University Ethics Committee and all experiments were conducted in accordance with the Declaration of Helsinki (protocol code: 1044396-202410-HR-171-01).

### 2.2. 3D-Printed Arch-Support Insoles

Each subject received customized 3D-printed foot orthoses designed to provide arch support and enhance stability. The process began with a 3D scanner (Shining 3D, San Leandro, CA, USA) to capture the foot’s shape. The SI was then designed by reverse-engineering techniques with Fusion 365 (Autodesk Inc., San Francisco, CA, USA). The design was customized to cover two-thirds of the foot length with the end of the SI positioned at approximately 10% of foot length behind the first metatarsal head [6]. The design included a 2.5 mm thick structure, modeled to the foot’s relaxed plantar surface, with a U-shaped top and flat bottom in the calcaneus area to enhance stability and minimize overpronation [6]. The SI was produced using Fused Deposition Modeling technology with a Polylactic acid filament, set to optimal parameters for durability and support. To ensure comfort, a polyamide nylon layer with 1 mm cushioning was added, providing both sufficient arch support and effective shock absorption during walking. The total time required to print each SI was approximately 4 h.

### 2.3. Experimental Procedures

Gait analysis data were collected in the Integrative Movement Science Laboratory at Gachon University. To gather kinematic and kinetic data, the following tools were used: Kinovea (version 2023.1.2) for ankle alignment analysis, OptoGait (Microgate, Bolzano, Italy) for gait pattern assessment, and F-Scan (Tekscan, Norwood, MA, USA) for center-of-force measurements. Video recordings using OptoGait were taken from two perspectives: the rear and side views. The cameras were positioned approximately 1.5 m away from the subject, with one aligned directly behind and the other perpendicular to the subject’s coronal plane. During the second visit, customized SIs were provided to ensure proper fitting on the plantar surface and to check for any manufacturing defects from the 3D printer. To minimize learning effects, subjects underwent a 5 min familiarization session with each condition, which was conducted 2 to 3 days before experimental trials. During the experimental trials, subjects performed 10% incline walking at a self-selected speed under two conditions: (1) walking with shoes only (shoe), (2) walking with the shoes equipped with 3D-printed arch-support insoles (SIs). To maintain consistency across all experiments, commercially available Converse-style sneakers shoes with a comfortable, flat outsole and a 2 cm height (JK company Inc., Gimhae, Republic of Korea) were used for all conditions. At the third visit, subjects participated in the two experimental conditions, which were performed on 10% incline on a treadmill in a permuted crossover design.

### 2.4. Gait Pattern Analysis

Spatiotemporal parameters of gait including stride length, cadence, and gait velocity were analyzed using the OptoGait Photoelectric Cell System (version 1.13.24.0). The OptoGait system consists of two 1 m bars, one for transmitting and the other for receiving, each equipped with 96 LED diodes spaced 1 cm apart and positioned 2 mm above the floor level on the side bars of a treadmill. Data were collected at a 1000 Hz sampling rate as the LED diodes were disrupted by foot movement during walking. Measurements were taken under an incline walking condition on the treadmill, enabling comparison of gait patterns between the experimental and control conditions. As reported in a previous study, insoles play a significant role in increasing the peak pressure in the metatarsal area while supporting the midfoot contact area, leading to notable changes in propulsive and foot-flat phases, respectively [11]. Based on the evidence, the current study was focused on the analysis of three key spatiotemporal parameters: the contact, foot-flat, and propulsive phases. These phases can provide critical insights into the biomechanical adaptations induced by the usage of customized arch-support insoles, particularly during uphill walking. The percentage contributions of each stance phase, as analyzed by the OptoGait system, were applied to the individual data to further assess the contribution of each phase to the center of force and ankle alignment in the subsequent analysis.

### 2.5. Center-of-Force (COF) Analysis

The COF displacement was measured using Tekscan F-scan systems (Tekscan Inc., Norwood, MA, USA) across at least 15 steps per trial. The F-scan system, a wireless plantar-pressure outsole, monitors the COF shifts at a sampling rate of 100 Hz at each step. The wired pressure outsole with the F-scan sensor (Model #3000E Tekscan, Inc., Boston, MA, USA) were placed below the shoe. The outsole is composed of 960 individual pressure-measuring sensors, which enables a detailed analysis of the COF dynamics during walking.

### 2.6. Ankle Alignment Analysis

Reflective markers (NaturalPoint Inc., Corvallis, OR, USA) were placed on the posterior talus, calcaneus, and mid-gastrocnemius of each subject for video analysis. Video cameras were set at 30 frames per second. The camera height was set at the ankle levels to ensure proper focus on the ankle joint. The final one minute of recording from a total of four minutes of walking under all conditions was selected for analysis. Ankle alignment during uphill walking was assessed by tracking pronation and supination movements using Kinovea software (version 2023.1.2), which provided a detailed analysis of the changes in ankle angles throughout the gait cycle [18]. From the rear view, the pronation angle was determined by drawing a line from the posterior talus to the midpoint of the gastrocnemius and calcaneus. Positive values represented pronation while negative values represented supination. A total of 10 consecutive gait cycles per foot were analyzed for each condition during the last minute and the mean values across the 10 gait cycles were utilized for further analysis.

### 2.7. Statistical Analyses

Regarding the sample size, the G-Power program (version 3.1, Universität Kiel, Kiel, Germany) was utilized to calculate the required sample size. The calculation was based on a large effect size (0.5), an alpha level of 0.05, and a statistical power of 0.08, resulting in a total of 21 participants, which is consistent with similar previous studies [6,19]. The sample size was considered adequate for detecting statistically significant differences between the experimental and control conditions. For all statistical analyses, GraphPad Prism (version 10.3.1, GraphPad Software, San Diego, CA, USA) was employed [20]. The normality of the data was verified using the D’Agostino–Pearson omnibus and Shapiro–Wilk tests. A two-way ANOVA followed by Sidak’s multiple comparisons test were performed to evaluate differences in gait patterns, COF, and joint angles between the experimental and control conditions. For specific comparisons of interest between the conditions, paired *t*-tests were conducted using Microsoft Excel (Microsoft Corporation, CA, USA) [21]. All data were presented as the mean ± standard error of the mean (SEM). A significance level of *p* < 0.05 was applied to all statistical tests.

## 3. Results

### 3.1. Gait Patterns

**Figure 1:**
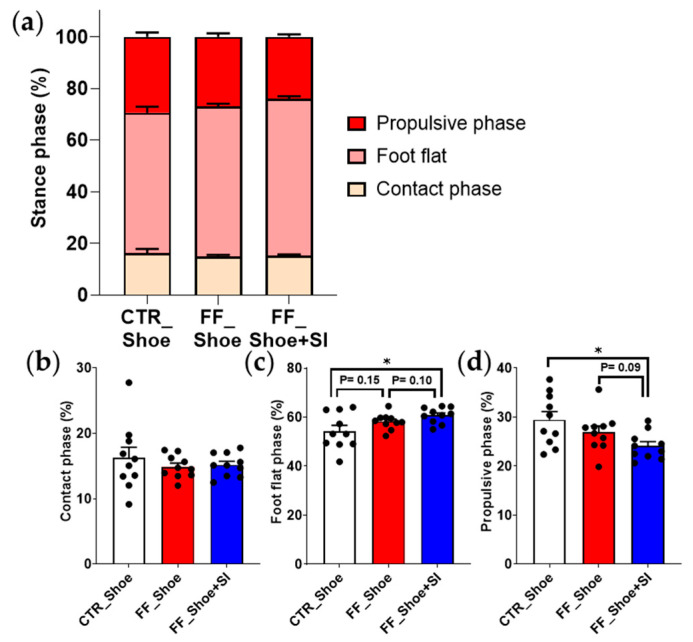
Gait pattern analysis in the comparison of the stance phase components. (**a**) Stance phase across conditions; (**b**) contact phase duration; (**c**) foot-flat phase duration; (**d**) propulsive phase duration. Significant main effects (*p* ≤ 0.05) were observed between CTR and FF groups in foot-flat and propulsive phases; * indicates significant differences between the indicated group. CTR: control, FF: flat foot.

The stance phase, divided into the contact, foot-flat, and propulsive phases, showed notable differences across conditions (Figure 1). In the FF group, the foot-flat phase duration under the shoe condition showed an increasing trend compared to the control group, with a statistically significant increase observed under the SI condition (*p* = 0.02). Additionally, while the SI condition in the FF group tended to prolong the foot-flat phase compared to the shoe condition (*p* = 0.10), this trend was not statistically significant. Importantly, the propulsive phase duration in individuals with FF with the SI was significantly reduced compared to the control group under the shoe condition (*p* = 0.01). Within the FF group, the SI conditions showed a decreasing trend compared to the shoe condition (*p* = 0.09), but the difference did not reach statistical significance.

### 3.2. Center of Force

**Figure 2:**
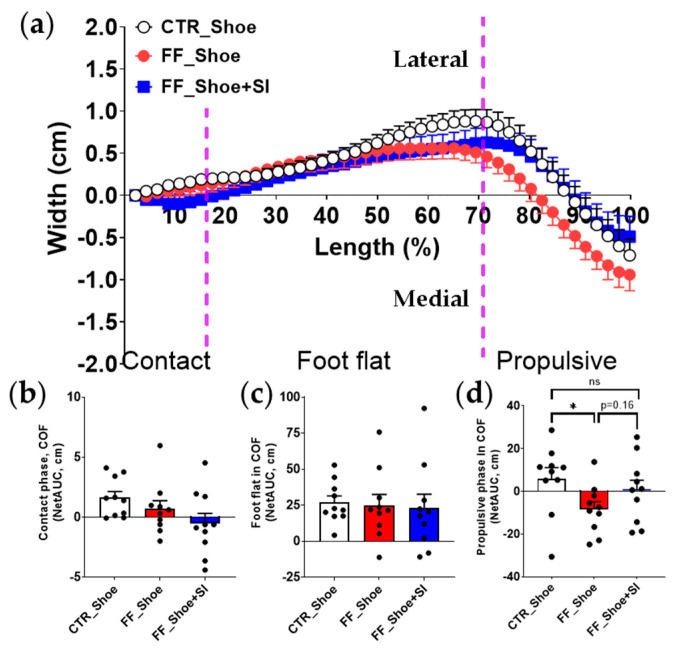
Average center-of-force path during uphill walking. (**a**) The average COF path from the initial contact to complete toe-off; (**b**) NetAUC of the average COF during the contact phase from initial contact to full foot support; (**c**) NetAUC of the average COF during the foot-flat phase, representing the duration when the foot is in complete contact with the ground; (**d**) NetAUC of the average COF during the propulsive phase from heel lift to complete toe-off. A significant main effect (*p* ≤ 0.05) was observed between CTR and FF groups in the propulsive phase; * and *ns* indicate significant and no significant differences between the indicated groups, respectively. Dash line represents an approximate boundary for the contact, foot-flat, and propulsive phases, determined based on the averaged phase distribution across participants. NetAUC: net area under the curve, CTR: control, FF: flat foot.

The average path of the COF revealed distinct improvements with the SIs in individuals with FF (Figure 2). Under the shoe condition, the COF during uphill walking in the FF group was significantly more medially shifted compared to the control group during the propulsive phase (*p* = 0.04). While the SI condition showed a tendency to rectify this medial deviation during the propulsive phase (*p* = 0.16), the improvement did not reach statistical significance. Nonetheless, the COF path under the SI condition in individuals with FF was more laterally aligned compared to the shoe condition, reducing the deviation observed in the FF group. Notably, the COF path under the SI condition closely aligned with the control shoe condition, and no significant differences were detected between these two conditions.

### 3.3. Ankle Joint Angle (Pronation vs. Supination)

**Figure 3:**
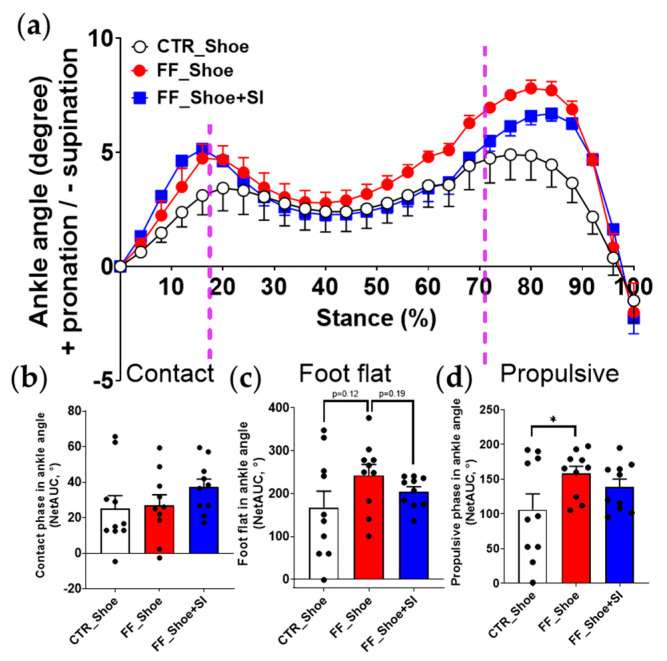
Ankle joint angles during uphill walking (**a**) The ankle joint angles from initial contact to complete toe-off; (**b**) NetAUC of ankle joint angles during the contact phase; (**c**) NetAUC of ankle joint angles during the foot-flat phase; (**d**) NetAUC of ankle joint angles during the propulsive phase. A significant main effect (*p* ≤ 0.05) was observed between CTR and FF groups in the propulsive phase; * indicates significant differences between the indicated groups. Dash line represents an approximate boundary for the contact, foot-flat, and propulsive phases, determined based on the averaged phase distribution across participants. NetAUC: net area under the curve, CTR: control, FF: flat foot.

Changes in the ankle joint pronation angle varied significantly across conditions (Figure 3). Overall, individuals with FF under the shoe condition exhibited greater pronation angles compared to both the SI condition and the control group under the shoe condition. During the foot-flat phase, the ankle joint pronation angle in individuals with FF under the shoe condition was greater compared to the control group (*p* = 0.12), but this difference was not statistically significant. The SI condition in individuals with FF mitigated the pronation angle compared to the shoe condition in the same group (*p* = 0.19), though this change also did not reach statistical significance. During the propulsive phase, the differences in ankle joint pronation angle between individuals with FF and the control group under the shoe condition became more pronounced (*p* = 0.05). Importantly, the SI condition in individuals with FF resulted in no significant differences compared to the control group under the shoe condition.

## 4. Discussion

The current study demonstrated that 3D-printed customized arch-support insoles significantly improved the gait mechanics in individuals with functional pes planus during uphill walking. Specifically, the SI condition corrected the medial deviation of the center of force observed in individuals with FF under the shoe condition, closer to the alignment seen in the control group under the shoe condition. Moreover, the SIs reduced overpronated angles during the stance phase, particularly in the foot-flat and propulsive phases, aligning ankle joint kinematics closer to those of the control group.

The reduction in propulsive phase duration observed with the SI condition aligns with previous findings suggesting that increased foot stability reduces compensatory mechanisms during propulsion [11,15]. Previous studies have highlighted that individuals with FF exhibit prolonged propulsive phases due to inefficient force transfer and instability during push-off [22,23]. The current results suggest that the improved rearfoot stability provided by the SIs minimizes compensatory adjustments, thereby shortening the propulsive phase (Figure 1). However, the duration of the propulsive phase with the SIs showed a different pattern compared to the control under the shoe condition, which is inconsistent with our hypothesis expecting a similar pattern to the control group. This discrepancy likely arises from the unique stabilizing properties of the SIs, which enhance midfoot support and promote supination, leading to extended engagement of the foot-flat phase. Supporting this notion, previous research has demonstrated that during uphill walking, arch-support insoles increased the contact area of the midfoot and the first toe-pushing ability [11], resulting in greater efficiency during propulsive phase after a given amount of time. Such biomechanical adaptations, while distinct from the control group, may ultimately optimize force transfer and push-off efficiency during uphill walking.

The SIs stimulated a lateral deviation in the COF during the propulsive phase, aligning it closely under the control shoe condition, while individuals with FF under the shoe condition exhibited a greater medial deviation (Figure 2). This finding is consistent with previous study showing that the foot orthoses induce a more lateral shifting in the average center of pressure [6]. The observed lateral deviation in the COF can be attributed to the stabilizing effects of the SIs, which support the medial longitudinal arch and redistributes plantar pressure away from the midfoot toward the lateral forefoot during the propulsive phase [11,15]. By providing structural support, the SI likely minimizes excessive pronation, thereby improving rearfoot stability and enabling a more efficient transfer of force through the lateral forefoot during the propulsive phase.

A key factor contributing to this stabilization is the design of the 3D-printed SI, which includes adequate rearfoot support in addition to the midfoot arch support. Unlike conventional orthoses that primarily focus on midfoot alignment [6,24], the SI used in this study incorporates a flat and stable calcaneal base, effectively reducing medial instability in the rearfoot. This design facilitates a balanced load distribution across the plantar surface, leading to a more lateral COF path in the propulsive phase. However, a discrepancy exists regarding the center-of-pressure path; earlier studies reported lateral shifts in the midfoot, whereas the current result showed lateral shift in the forefoot. This discrepancy may be due to the differences in experimental conditions including level walking vs. uphill walking. Uphill walking requires increased plantar flexion and alters the mechanics of the push-off phase [11], potentially emphasizing the role of lateral forefoot support. The unique design of the 3D-printed SIs in the current study, which combines arch and heel support, may also account for the observed deviations in COF alignment compared to conventional orthoses. Moreover, the unique design of the SIs can provide a biomechanical advantage by optimizing foot mechanics under increased load and incline-specific demands. Clinical application of such designs may help mitigate overpronation and associated joint stress in individuals with FF. Future orthotic designs should incorporate customization techniques and material innovations that enhance biomechanical efficiency in diverse walking conditions, such as uneven or inclined grounds.

In ankle angle analysis, a distinct pattern in ankle angle compared to the COF was observed. The SI reduced pronation of the ankle joint to the extent that there was no significant difference from the control shoe condition, while individuals with FF under the shoe condition exhibited greater pronation compared to the control group (Figure 3). In line with the current finding, previous studies reported that foot orthoses reduced ankle eversion (e.g., pronation) during the middle stance phase (approximately 20–80%) by stabilizing the rearfoot and controlling medial collapses [7,24]. However, these studies also showed results that differ from the current study, particularly in the late stance phase (approximately 80–100%). This discrepancy could be attributed to differences in orthotic design, such as variations in the heel support structure [9] or the usage of wedges with different thicknesses [7]. Additionally, the increased plantar flexion demands during uphill walking could alter the SI’s effects in the propulsive phase by shifting the mechanics of push-off.

The foot-flat phase results revealed mixed findings with the SI condition showing trends toward improved ankle alignment but no difference in the COF. This ambiguity aligns with prior research reporting limited effects of orthoses on overall stance duration, suggesting that variability may be influenced by factors such as walking speed [25], or incline gradient [15,25] during the gait cycle. Despite the lack of statistical significance, the observed trends still indicate the potential benefits of the SIs in enhancing stability during the foot-flat phase, warranting further investigation with larger sample sizes. Regarding the long-term application of 3D-printed customized insoles, Ribeiro et al. recently demonstrated that using insoles for 6 months significantly improved calcaneus pain, plantar load, and foot function, including walking difficulty and the 6 min walking distance, compared to a shoe-alone group in women with plantar fasciitis [26]. Additionally, factors such as body mass composition during childhood and adolescence can play a critical role in the development and progression of flat foot into adulthood [27]. Thus, it is highly plausible that the long-term usage of 3D-printed customized SIs may not only help alleviate plantar pain and fatigue but also contribute to improved foot mechanics and functional mobility in individuals with FF, emphasizing its potential as an effective intervention for managing flat-foot-related impairments.

This study has several limitations. First, the study focused exclusively on uphill walking, and the findings may not be generalizable to other environmental contexts, such as uneven or downhill walking. Second, while the 3D-printed SIs were customized for each participant, variations in material properties and individual biomechanics may have influenced the results. Finally, the study did not account for long-term adaptation to orthotic use, which could yield different outcomes over extended periods.

## 5. Conclusions

In conclusion, this study highlights the potential benefits of 3D-printed customized arch-support insoles in improving gait mechanics and ankle alignment in young adults with functional pes planus during uphill walking. The SIs effectively reduced the medial COF deviation and mitigated excessive pronation angles during the propulsive phase, demonstrating the therapeutic effectiveness of 3D-printed customized SIs in ameliorating gait impairments associated with FF. However, further research is needed to investigate the long-term effects of SI usage on functional outcomes, its applicability across various walking conditions, and optimization of the design to enhance efficacy. These efforts will deepen the understanding of the clinical potential of 3D-printed orthoses.

## Data Availability

The data presented in this study are available on request from the corresponding author. The data are not publicly available due to ethical restrictions.

## References

[1] Harris R.I., Beath T. (1948). Hypermobile flat-foot with short tendo achillis. J. Bone Jt. Surg. Am..

[2] Tareco J.M., Miller N.H., MacWilliams B.A., Michelson J.D. (1999). Defining flatfoot. Foot Ankle Int..

[3] Huang Y.-C., Wang L.-Y., Wang H.-C., Chang K.-L., Leong C.-P. (2004). The Relationship between the Flexible Flatfoot and Plantar Fasciitis: Ultrasonographic Evaluation. Chang Gung Med. J..

[4] Michelson J.D., Durant D.M., McFarland E. (2002). The injury risk associated with pes planus in athletes. Foot Ankle Int..

[5] Tong J.W.K., Kong P.W. (2013). Association between foot type and lower extremity injuries: Systematic literature review with meta-analysis. J. Orthop. Sports Phys. Ther..

[6] Lin K.-W., Chou L.-W., Su Y.-T., Wei S.-H., Chen C.-S., Lin K.-W., Chou L.-W., Su Y.-T., Wei S.-H., Chen C.-S. (2021). Biomechanical Effect of 3D-Printed Foot Orthoses in Patients with Knee Osteoarthritis. Appl. Sci..

[7] Hsu C.Y., Wang C.S., Lin K.W., Chien M.J., Wei S.H., Chen C.S. (2022). Biomechanical Analysis of the FlatFoot with Different 3D-Printed Insoles on the Lower Extremities. Bioengineering.

[8] Cheung J.T.M., Zhang M. (2005). A 3-dimensional finite element model of the human foot and ankle for insole design. Arch. Phys. Med. Rehabil..

[9] Li L., Yang L., Yu F., Shi J., Zhu L., Yang X., Teng H., Wang X., Jiang Q. (2018). 3D printing individualized heel cup for improving the self-reported pain of plantar fasciitis. J. Transl. Med..

[10] Morio C., Lake M.J., Gueguen N., Rao G., Baly L. (2009). The influence of footwear on foot motion during walking and running. J. Biomech..

[11] Huang Y.P., Peng H.T., Wang X., Chen Z.R., Song C.Y. (2020). The arch support insoles show benefits to people with flatfoot on stance time, cadence, plantar pressure and contact area. PLoS ONE.

[12] Shin J.Y., Ryu Y.U., Yi C.W. (2016). Effects of insoles contact on static balance. J. Phys. Ther. Sci..

[13] Schmitt A.P.L., Liebau K.H., Hamm A., Hacke C., Mittelmeier W., Schulze C. (2022). Comparison of the influence of supportive and sensorimotor insoles in the muscle activity of tibialis anterior and peroneus longus in combat boots. Foot.

[14] Peng Y., Wong D.W.C., Wang Y., Chen T.L.W., Tan Q., Chen Z., Jin Z., Zhang M. (2020). Immediate Effects of Medially Posted Insoles on Lower Limb Joint Contact Forces in Adult Acquired Flatfoot: A Pilot Study. Int. J. Environ. Res. Public Health.

[15] Jiang Y., Yang J., Tian H., Jiang C., Wang H. (2024). Comparative study of the effects of custom-made insole and ordinary insole in adults with flexible flatfoot on different slopes. Technol. Health Care.

[16] Yang Z., Liu F., Cui L., Liu H., Zuo J., Liu L., Li S. (2020). Adult rigid flatfoot: Triple arthrodesis and osteotomy. Medicina.

[17] Kirmizi M., Sengul Y.S., Angin S. (2022). The effects of calf muscles fatigue on dynamic plantar pressure distribution in normal foot posture and flexible flatfoot: A case-control study. J. Back Musculoskelet. Rehabil..

[18] Iskandar M.N.S., Loh R.B.C., Ho M.Y.M., Pan J.W., Kong P.W. (2023). Crossover gait in running and measuring foot inversion angle at initial foot strike: A front-view video analysis approach. Front. Bioeng. Biotechnol..

[19] Shi L., Ye X., Han D., Yang C., Tu Y. (2023). Acute Effects of Back Squat Combined with Different Elastic Band Resistance on Vertical Jump Performance in Collegiate Basketball Players. J. Sports Sci. Med..

[20] D’Antona G., Ragni M., Cardile A., Tedesco L., Dossena M., Bruttini F., Caliaro F., Corsetti G., Bottinelli R., Carruba M.O. (2010). Branched-chain amino acid supplementation promotes survival and supports cardiac and skeletal muscle mitochondrial biogenesis in middle-aged mice. Cell Metab..

[21] Park S., Jang J., Choi M.D., Shin Y.-A., Schutzler S., Azhar G., Ferrando A.A., Wolfe R.R., Kim I.-Y. (2020). The Anabolic Response to Dietary Protein Is Not Limited by the Maximal Stimulation of Protein Synthesis in Healthy Older Adults: A Randomized Crossover Trial. Nutrients.

[22] Buldt A.K., Murley G.S., Butterworth P., Levinger P., Menz H.B., Landorf K.B. (2013). The relationship between foot posture and lower limb kinematics during walking: A systematic review. Gait Posture.

[23] Levinger P., Murley G.S., Barton C.J., Cotchett M.P., McSweeney S.R., Menz H.B. (2010). A comparison of foot kinematics in people with normal- and flat-arched feet using the Oxford Foot Model. Gait Posture.

[24] Lin K.W., Hu C.J., Yang W.W., Chou L.W., Wei S.H., Chen C.S., Sun P.C. (2019). Biomechanical Evaluation and Strength Test of 3D-Printed Foot Orthoses. Appl. Bionics Biomech..

[25] Waheed J., Arora N.K., Khan M.H. (2022). Comparison of Leg Muscle Activity During Level and Uphill Walking in Individuals with Flat Foot and Normal Foot: A Cross-Sectional Study. Polish J. Sport Tour..

[26] Longo G., Denaro V., Ribeiro A.P., Maria S., João A. (2022). The Effect of Short and Long-Term Therapeutic Treatment with Insoles and Shoes on Pain, Function, and Plantar Load Parameters of Women with Plantar Fasciitis: A Randomized Controlled Trial. Medicina.

[27] Aniśko B., Siatkowski I., Wójcik M. (2024). Body mass composition analysis as a predictor of overweight and obesity in children and adolescents. Front. Public Health.

